# Electrolyte Effects in Membrane‐Electrode‐Assembly CO Electrolysis

**DOI:** 10.1002/anie.202501505

**Published:** 2025-03-27

**Authors:** Qiucheng Xu, Bjørt Óladóttir Joensen, Nishithan C. Kani, Andrea Sartori, Terry Willson, John R. Varcoe, Luca Riillo, Anna Ramunni, Jakub Drnec, Ib Chorkendorff, Brian Seger

**Affiliations:** ^1^ Surface Physics and Catalysis (SurfCat) Section Department of Physics Technical University of Denmark Kongens Lyngby 2800 Kgs. Denmark; ^2^ Laboratory of Inorganic Synthesis and Catalysis Institute of Chemical Sciences and Engineering Ecole Polytechnique Fédérale de Lausanne (EPFL) Lausanne CH‐1015 Switzerland; ^3^ Experimental Division European Synchrotron Radiation Facility Grenoble 38000 France; ^4^ Department of Chemistry The University of Surrey Guildford GU2 7XH UK; ^5^ Industrie De Nora S.p.A. Via Leonardo Bistolfi 35 Milan 20134 Italy

**Keywords:** Anodic oxidation, CO electrolysis, Electrocatalysis, Electrolyte effect, Membrane‐electrode assembly

## Abstract

Membrane‐electrode‐assembly (MEA)‐based CO electrolysis (COE) has demonstrated the capability to produce C_2+_ products with high faradaic efficiency at ampere‐level current densities. However, most studies on COE have achieved performance benchmarks under strongly alkaline conditions (e.g., ≥1 m KOH, pH ≥14), raising the question of whether such high pH levels are essential for optimal performance. In this study, we investigated the effects of different electrolytes (KHCO_3_, K_2_CO_3_, and KOH) on MEA‐based CO electrolysis, focusing on the influence of pH and the impact of anodic oxidation on the selectivity of various liquid products. By adjusting electrolyte concentration and pH, we achieved significant partial current densities for ethanol (189 ± 5 mA cm^−2^) and propanol (89 ± 4 mA cm^−2^) using 0.5 M K_2_CO_3_. This high performance is attributed to the creation of a moderate local alkaline environment and the relatively high resistance to anodic oxidation. Additionally, durability measurements emphasized the critical importance of eliminating anodic oxidation to optimize MEA‐based COE for ethanol and propanol production.

## Graphical abstract

Membrane‐electrode assembly‐based CO electrolysis (COE) has shown the ability to produce C_2_+ products with high faradaic efficiency at ampere‐level current densities. This study explored the effects of different electrolytes (KHCO_3_, K_2_CO_3_, and KOH) on COE, focusing on pH and anodic oxidation. Using 0.5 m K_2_CO_3_, we achieved high partial current densities for ethanol and propanol, highlighting the importance of minimizing anodic oxidation.

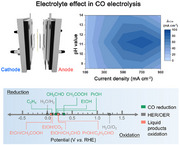

## Introduction

Electrochemical synthesis of chemical commodities through CO electrolysis presents a promising pathway for leveraging sustainable electricity sources.^[^ ^1^, ^2^, ^3^ ^]^ To date, membrane‐electrode assembly (MEA)‐based CO electrolysis (COE) has demonstrated the ability to produce C_2+_ products (e.g., ethylene, acetate, ethanol, and propanol) with high faradaic efficiency at ampere‐level current densities.^[^ ^4^, ^5^, ^6^, ^7^ ^]^ Advancing COE technology will require focusing on maximizing the faradaic efficiency (FE) toward specific target products. Given the relative ease of separating gas and liquid products, managing COE selectively to favor ethylene production alongside a chosen liquid product, such as acetate, ethanol, or propanol, is a realistic and impactful objective.

Advancements in material design and surface modification have been instrumental in enhancing the selectivity of CO reduction.^[^ ^8^, ^9^, ^10^, ^11^, ^12^, ^13^ ^]^ For instance, Wang et al. synthesized a novel Ag─Ru─Cu catalyst, achieving a high FE of 36% ± 3% for n‐propanol at 300 mA cm^−2^ by stabilizing key intermediates (*OCCO) and promoting CO adsorption.^[^ ^10^ ^]^ In another study, a Cu/Cu_2_O‐derived nanosheet catalyst modified with a hydrophobic n‐butylamine layer demonstrated high ethanol selectivity, reaching an FE of 69% ± 3% at 160 mA cm^−2^.^[^ ^9^ ^]^ Additionally, operating conditions significantly influence product composition in MEA systems. Jiao and colleagues leveraged anodic oxidation to in situ convert liquid products of ethanol into acetate during COE.^[^ ^14^, ^15^ ^]^ By tailoring anodic catalysts and anion exchange membranes, they achieved an acetate stream with a purity of >97% at 200 mA cm^−2^ in the anolyte.

However, the majority of studies on COE, including those mentioned above, have achieved performance benchmarks under strongly alkaline conditions (e.g., ≥1 m KOH), raising the question of whether such a high pH is necessary for producing all types of COE products. While a few fundamental studies have explored electrolyte effects including pH and cation influence in H‐cell or flow‐cell systems,^[^ ^16^, ^17^, ^18^ ^]^ their insights cannot always be directly translated to MEA systems, where local pH and cation concentrations are strongly influenced by operating conditions and MEA design.^[^ ^4^, ^19^, ^20^ ^]^ Therefore, investigating electrolyte effects within MEA‐based CO electrolyzers has emerged as an important research direction.

In this study, we investigated the electrolyte effects in MEA‐based COE, focusing on pH influence and the impact of anodic oxidation on the selectivity of liquid products. By adjusting the electrolyte, we achieved significant partial current densities for ethanol (189 ± 5 mA cm^−2^) and propanol (89 ± 4 mA cm^−2^) at 0.5 m K_2_CO_3_. Furthermore, by leveraging anodic oxidation, a high acetaldehyde proportion (≈28%) was obtained in the anolyte during stability testing.

## Results and Discussion

### Electrolyte Effect on COE Overall Performance

For this study, three electrolytes including KHCO_3_, K_2_CO_3,_ and KOH were used as the anolyte for COE, each with a cation concentration maintained at 1 m, but varying bulk pH values ranging from 8.5 to 14. In the designed experiment, the bulk K^+^ concentration in the electrolyte was controlled to remain consistent, enabling a focused investigation into the influence of pH on performance. Typically, applying a higher current density at the cathode results in the formation of a stronger local alkaline environment.^[^ ^21^ ^]^ Adjusting the bulk electrolyte pH varies the pH gradient across the catalyst layer, thereby influencing the local pH close to the electrode surface and thus the product selectivity.

**Figure 1:**
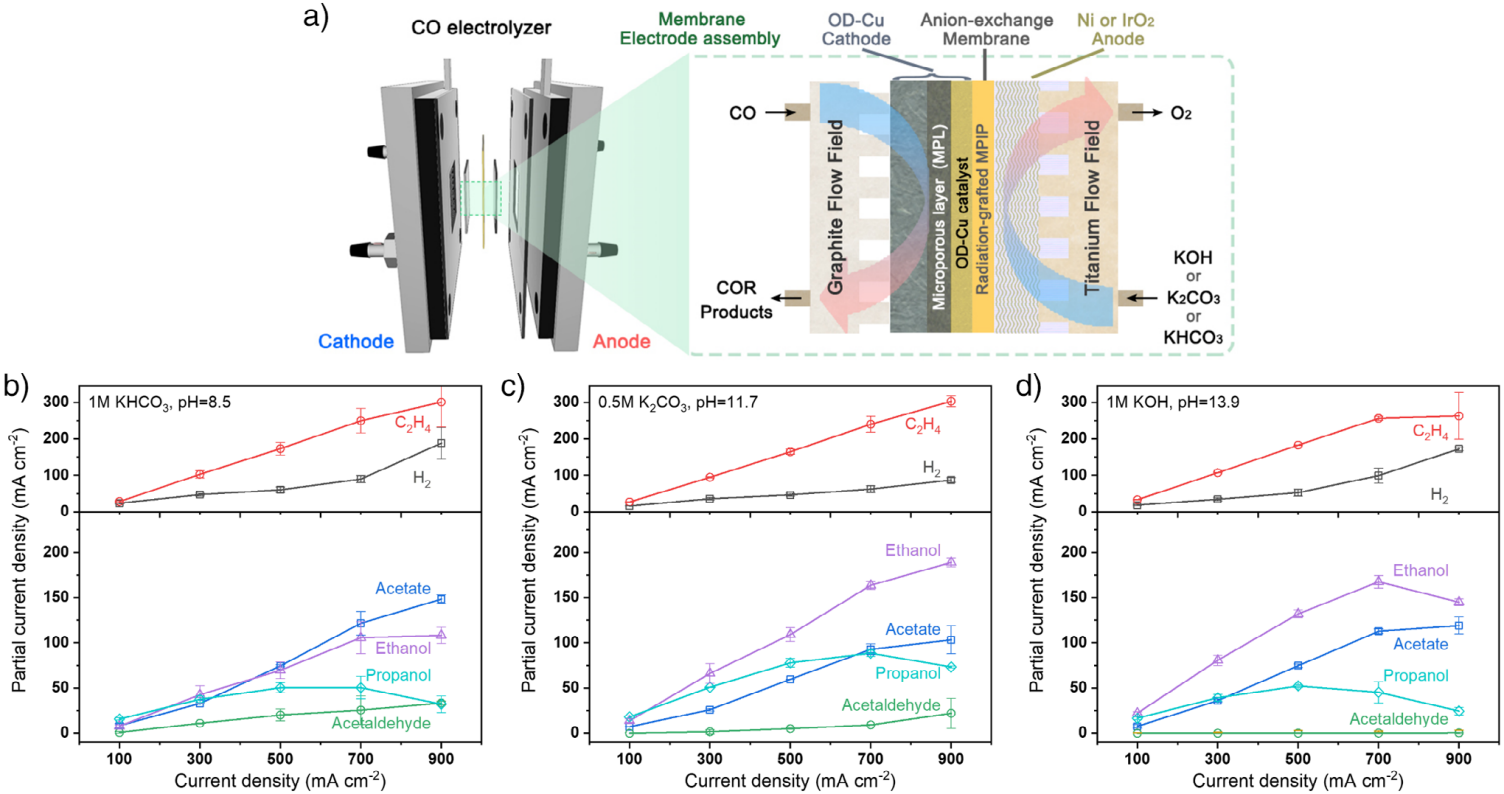
a) Schematic illustration of the CO electrolyzer and its MEA configuration. Partial current densities of different products (including hydrogen, ethylene, acetaldehyde, acetate, ethanol, and propanol) when operating COE in b) 1 m of KHCO_3_, c) 0.5 m of K_2_CO_3_ and d) 1 m of KOH.

Figure 1a presents a schematic illustration of the CO electrolyzer and the detailed configuration of its MEA. The digital image of the customized electrolyzer is shown in Figure S1. A radiation‐grafted, customized anion‐exchange membrane (AEM) containing N‐methyl‐piperidinium headgroups, referred to as RG‐MPIP, was utilized and placed between the cathode and anode. Oxide‐derived copper (OD‐Cu) was used as a benchmark cathode catalyst for all measurements. The XRD pattern and SEM images of pristine OD‐Cu are provided in Figures S2 and S3. Nickel foam or IrO_2_ on titanium felt was used as an anode for alkaline electrolytes (KOH and K_2_CO_3_) or neutral electrolytes (KHCO_3_), respectively. According to the Pourbaix diagram, Ni is unstable under electrochemical oxidation in neutral electrolytes and may contaminate the cathode, prompting the use of IrO_2_ as an alternative.

For the performance evaluation, variable current densities were applied from 100 to 900 mA cm^−2^. Figure S4 shows the flow diagram of our testing setup. A large volume of anolyte (200 mL) was provided to prevent pH changes during the measurement.^[^ ^22^ ^]^ Liquid products were collected from both the cathodic water trap and the anolyte for calculating the FEs. During COE, the OD‐Cu catalyst was rapidly reduced to metallic Cu within 1 min at a current density lower than 100 mA cm^−2^, as observed by in‐operando wide‐angle X‐ray scattering (Figure S5).

The partial current densities of gas and liquid products at different operating conditions are shown in Figure 1b–d and their corresponding FEs are given in Figure S6. The FE of O_2_ at the anode is provided in Figure S7. These results reflect the combined performance of both the cathode and anode. They show the hydrogen evolution reaction (HER) is suppressed better in the K_2_CO_3_ electrolyte, with its FE limited to below 10% even at a current density of 900 mA cm^−2^. Furthermore, while the partial current density of C_2_H_4_ showed a similar increasing trend across different electrolytes with increasing applied current density, the ability to produce liquid products varied significantly. Notably, the largest partial current densities for ethanol and propanol were achieved in the K_2_CO_3_ electrolyte at a pH of ≈11.7, reaching maximum values of 189 ± 5 mA cm^−2^ for ethanol and 89 ± 4 mA cm^−2^ for propanol. In contrast, the KHCO_3_ electrolyte at a pH of ≈8.5 exhibited relatively higher partial current densities for acetaldehyde and acetate. Previous theoretical studies suggest that *HCCO is the key intermediate determining selectivity, where oxygen protonation produces ethylene, and terminal carbon protonation forms oxygenate (acetate, acetaldehyde, ethanol, propanol).^[^ ^23^ ^]^ Therefore, separating liquid from gas products highlights clearer trends of electrolyte effects. Figure S8 illustrates the distribution of liquid products as a function of current density. When excluding the selectivity for gas products, acetaldehyde is most favored in KHCO_3_, ethanol in KOH, and propanol in K_2_CO_3_. Additionally, the energy efficiency (EE) of COE at different electrolytes is shown in Figure S9. Higher EE is achieved at lower current densities, and the EE follows the trend: K_2_CO_3_ > KOH > KHCO_3_ across all current density ranges. Given that K_2_CO_3_ also provides the highest selectivity for ethanol and propanol, these EE results further suggest that it is a promising electrolyte for CO electrolysis.

**Figure 2:**
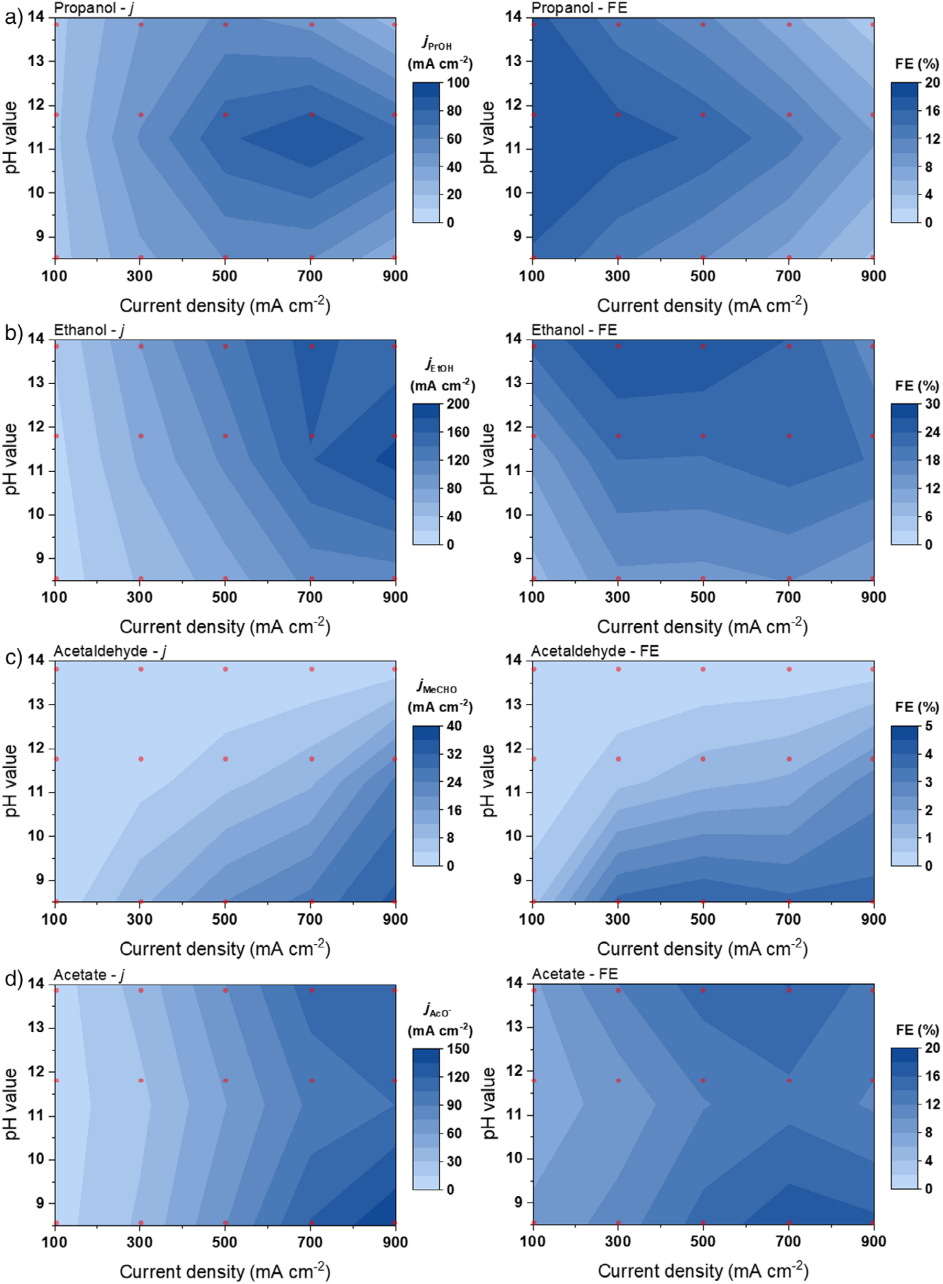
Partial current densities (left panel) and FEs (right panel) of a) propanol, b) ethanol, c) acetaldehyde, and d) acetate in terms of pH value of 1 m potassium electrolyte (3 values) and the applied current densities (5 values) by using the data from Figure 1. The red points represent the values used for plotting.

To provide a clear comparison, Figure 2 illustrates the production rate trends for various liquid products in terms of their partial current densities (left panel) or faradaic efficiencies (FEs, right panel) as a function of electrolyte pH values and applied current densities. Partial current densities represent intrinsic activity for specific products, while FEs highlight the selectivity under specific operating conditions, accounting for the effects of mass transfer limitation.^[^ ^24^ ^]^ As a result, differences are observed between their respective heatmap plots. Figure 2a shows that propanol production is favored under mildly alkaline conditions (K_2_CO_3_, pH ≈11.5). The highest FE for propanol (≈18%) is achieved at a low current density of 100 mA cm^−2^. The higher propanol selectivity observed at low current densities (i.e., low overpotential) under mildly alkaline conditions highlights the necessity of a moderated local alkaline environment. This environment enables a balance between achieving optimal coverage of C_1_ intermediates (*CO) and C_2_ intermediates (e.g., *CH_3_CHO, *HCCH) on the catalyst surface, facilitating C_3_ coupling.^[^ ^2^, ^25^ ^]^ However, the maximum partial current density for propanol is still achieved in K_2_CO_3_ at 700 mA cm^−2^, highlighting its advantage in optimizing reaction conditions for propanol formation.

For ethanol (Figure 2b), the heatmap indicates that higher partial current densities and selectivity are achieved either in KOH (strongly alkaline conditions) at moderate current densities or in K_2_CO_3_ (mildly alkaline conditions) at high current densities. Both conditions correspond to a moderate local alkaline environment, underscoring the importance of creating suitable local reaction conditions for ethanol production. In addition, acetaldehyde (Figure 2c) is favored under neutral conditions at higher current densities, while acetate (Figure 2d) is produced at larger current densities and is favored under both strongly alkaline and neutral conditions. Our previous studies indicate that the selectivity of liquid products in a zero‐gap MEA system is influenced not only by the cathodic reaction but also significantly by the anodic reaction.^[^ ^23^, ^26^ ^]^ In this system, the in situ generated liquid products can cross from the cathode to the anode, leading to unintended anodic oxidation. Despite employing strategies in our experiments, such as using a customized AEM, a large anolyte volume, and a short testing period, to mitigate crossover and anodic oxidation, completely eliminating these issues remains challenging, particularly at higher current densities where product concentrations at both sides of the electrode‐membrane interface increase significantly. Consequently, the observed performance trends across different electrolytes cannot be solely attributed to pH differences. Therefore, a more detailed discussion of these phenomena is provided in the following section.

### Electrolyte Effect on Anodic Oxidation

To provide a clearer explanation of the observed selectivity changes, it is necessary to decouple the contributions of cathodic and anodic performance. Normally, the crossover of the liquid products is based on the property of the ion‐exchange membrane and the operating conditions. In our study, the customized RG‐MPIP (Figure S10) was utilized, which has a reduced diffusion coefficient due to its weaker swelling, compared to the commonly used commercial AEM (e.g., Sustainion).^[^ ^15^, ^22^ ^]^ However, even with mitigated diffusion, liquid products must exit the cathode either through evaporation or as micro‐liquid bubbles, which are then pushed to a collector. Evaporation is inefficient at room temperature, and micro‐liquid bubbles are not consistent scalable, resulting in low liquid product collection at the cathode. Benefiting from collecting liquid products from the cathode water trap and the anolyte reservoir, the differences between these allowed for the decoupling of anodic oxidation effects from the cathodic reduction. Acetate and acetaldehyde were only observed at the anode. The crossover ratios of ethanol and propanol, as shown in Figure S11, exhibit a consistent trend of decreasing ratios with increasing current density. Across all current densities, the crossover ratios follow the general trend: KOH > K_2_CO_3_ > KHCO_3_. We hypothesize that this behavior is influenced by membrane swelling in different electrolytes with varying pH.

**Figure 3:**
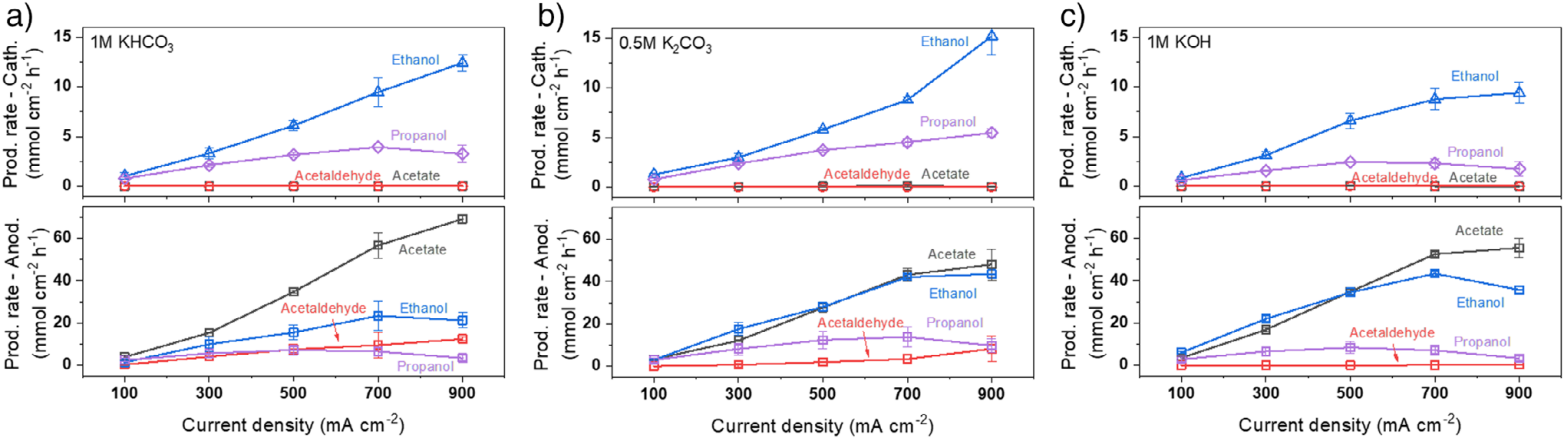
Production rate of four liquid products (including acetaldehyde, acetate, ethanol, and propanol) that were collected at the cathode (upper panel) and anode (lower panel) respectively when operating COE in a) 1 m of KHCO_3_, b) 0.5 M of K_2_CO_3_ and c) 1 m of KOH at variable applied current densities. Separate the combined liquid product results shown in Figure 1.

Figure 3 illustrates the respective production rates of liquid products at the cathode and anode across different electrolytes. Four primary liquid products including acetaldehyde, acetate, ethanol, and propanol were analyzed. While all four products were detected at the anode (lower panel of Figure 3), only ethanol and propanol were measured at the cathode (upper panel of Figure 3). Acetate, as an anion, rapidly crosses the AEM and is therefore only measurable at the anode.^[^ ^19^ ^]^ Acetaldehyde, with its low boiling point (≈20 °C), should theoretically be detectable at the cathode; however, it was not observed there. We speculate that this phenomenon originates from the high reactivity of acetaldehyde at the cathodic interface, resulting in a relatively low concentration that falls below the detection limit at the cathode side. During electrolysis, acetaldehyde may 1) rapidly diffuse with water to the anode, where it can be maintained and measured in the anolyte; 2) remain at the cathode interface, where it can either be further reduced to ethanol or react with OH⁻ in the strong local alkaline environment to form acetate.^[^ ^27^ ^]^ Even if some acetaldehyde is collected in the water trap at the cathode, its concentration may be below the detection limit. These factors create significant uncertainties, making the detection of acetaldehyde at the cathode side challenging. At the anode side, a relatively high production rate of acetaldehyde was detected in 1 m KHCO_3_ (Figure 3a), with its rate increasing with applied current density. For 0.5 m K_2_CO_3_ (Figure 3b), a lower production rate of acetaldehyde was observed at the anode, but it increased with current density. In contrast, almost no acetaldehyde was observed at the anode under 1 m KOH (Figure 3c).

A further comparison of ethanol reveals that production rates at the cathode consistently increased with current density, whereas this trend was not observed at the anode. Meanwhile, the production rate of propanol at both electrodes showed a slight mismatch, with maximum peaks in current density appearing earlier at the anode. The above results suggest significant ethanol oxidation and slight propanol oxidation at the anode during COE, which are closely correlated with discrepancies in acetaldehyde formation across various electrolytes, likely due to differences in anodic oxidation under varying pH conditions.

**Figure 4:**
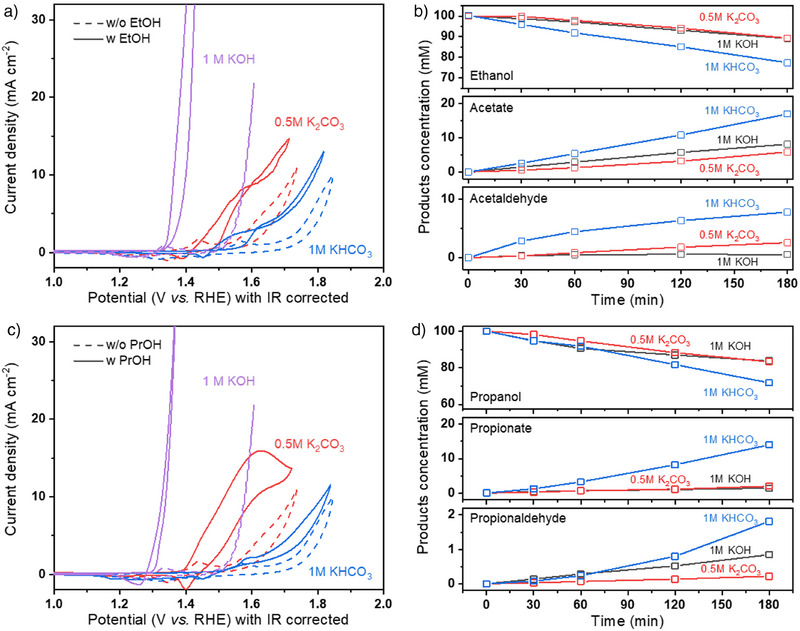
Cyclic voltammetry (CV) curve of nickel catalyst in different electrolytes with and without the addition of a) 1 m ethanol (EtOH) and c) 1 M n‐propanol (PrOH). Concentration changes of liquid products during water electrolysis in different electrolytes by adding b) 0.1 m ethanol or d) 0.1 m n‐propanol at 500 mA cm^−2^ over a 3 h measurement period.

To further investigate the oxidation trends and product distribution for ethanol oxidation and propanol oxidation in different electrolytes, several electrochemical oxidation control experiments were conducted. Figure 4a,c shows the CV curves of a nickel anode catalyst in 1 m KHCO_3_, 0.5 m K_2_CO_3_, and 1 m KOH, both with and without the addition of ethanol or propanol. Adding ethanol or propanol significantly shifts the CV curves to more cathodic potentials for all three electrolytes. This shift is attributed to the more cathodic equilibrium potentials of ethanol and propanol oxidation (<0.25 V vs RHE) compared to water oxidation (1.23 V vs RHE).

Further oxidation product analysis was performed during water electrolysis experiments, in which 100 mM ethanol or propanol was added to 1 m KHCO_3_, 0.5 m K_2_CO_3_, and 1 m KOH as the anolyte. During electrolysis, the cathode was flowed with Ar, and the anolyte was recycled. Samples of the anolyte were collected at different intervals to monitor changes in liquid product concentrations. In this setup, ethanol or propanol oxidation competes with water oxidation, simulating the anodic conditions encountered during COE. Figure 4b shows that ethanol converts to acetate in all electrolytes and to acetaldehyde in KHCO_3_ and K_2_CO_3_ but not in KOH. While it appears that ethanol oxidation to acetaldehyde is favored in neutral conditions, this trend may also be influenced by acetaldehyde's chemical stability, which differs across electrolytes. Our recent study demonstrated that acetaldehyde is stable in neutral conditions (KHCO_3_) but not in alkaline conditions (KOH).^[^ ^27^, ^28^ ^]^ Previous literature also highlights that the observed products depend not only on reaction pathways but also on the influence of electrolyte pH on the stability and reactivity of intermediates.^[^ ^29^ ^]^ We hypothesize that ethanol is oxidized to acetaldehyde to a minor extent in alkaline electrolytes but is quickly converted to acetate, or that acetaldehyde exists in various hydrated, aggregated, or polymerized states in alkaline solution via aldol or Tishchenko reactions.^[^ ^28^ ^]^ Ethanol conversion ratios further show that 23% of ethanol was oxidized in 1 m KHCO_3_ after 180 min, compared to only 11% in 0.5 m K_2_CO_3_ and 1 m KOH. Figure 4d further shows that propionate is the main product of n‐propanol oxidation, with a small amount of propionaldehyde also formed, consistent with the literature.^[^ ^30^ ^]^ The most significant conversion occurred in 1 m KHCO_3_, consistent with trends observed in ethanol oxidation. These results suggest that ethanol and propanol oxidation exhibit less competitiveness compared to water oxidation under more alkaline conditions.

Additionally, the effect of K_2_CO_3_ electrolyte concentration on COE performance was investigated by comparing 0.05, 0.2, and 0.5 m, as shown in Figure S12. Minimal differences were observed as a function of electrolyte concentration, though a slight shift toward more oxidized products (acetate and acetaldehyde) was noted at lower concentrations and higher current densities (>700 mA cm^−2^), suggesting that anodic oxidation becomes more pronounced under these conditions.

Nevertheless, anodic oxidation is also highly influenced by various operational conditions, including anolyte flow rate, anode catalyst selection, membrane selection, and others. A higher anolyte flow rate dilutes crossover products and prevents their accumulation at the anode, thereby reducing oxidation.^[^ ^14^ ^]^ The choice of anode catalyst is also critical; compared to NiFeO_x_ anodes, IrO_x_ anodes exhibit minimal alcohol oxidation activity and are better suited for completely suppressing liquid product oxidation.^[^ ^14^ ^]^ Additionally, low‐permeability membranes effectively reduce ethanol crossover, suppressing its oxidation and preserving its concentration in the catholyte.^[^ ^15^ ^]^ Optimizing these parameters can collectively enhance the stability and selectivity of CO electrolysis by minimizing unwanted anodic oxidation.

To gain further insights, we used the knowledge obtained from these designed experiments to better understand and then correct the as‐achieved COE performance. In our case, since only trace amounts (within 1%) of propionate were observed, we conclude that propanol oxidation during COE is negligible. The weak propanol oxidation could be explained by its competition with ethanol oxidation. Compared to propanol, ethanol is produced in greater quantities during CO electrolysis and has a smaller molecular size, resulting in a higher crossover rate. This leads to a higher local concentration at the anode, which accelerates anodic reactions. This likely contributes to the inhibition of propanol oxidation relative to ethanol oxidation. Consequently, the propanol performance trend in Figure 2a can be considered reliable. Weakly alkaline conditions are favorable for propanol formation achieving high selectivity at low current densities (i.e., low overpotential region) and the highest production rate at an optimized current density of ≈700 mA cm^−2^. Low current densities allow for high CO coverage, which helps in coupling with C_2_ intermediates to produce C_3_ products.^[^ ^2^, ^25^ ^]^ As the current density increases, the coverage of *CO decreases due to the formation of more C_2_ intermediates, which favors the continued reduction of C_2_ intermediates to C_2_ products while inhibiting C_3_ coupling. Thus, the C_3_ products to C_2_ products ratio decreases as current density is increased, which is most prominently seen when comparing propanol to ethanol at the cathode in Figure 3.

In contrast, ethanol performance is significantly influenced by anodic oxidation, as indicated by the notable formation of acetaldehyde and acetate. Some strategies to mitigate anodic oxidation have been proposed in our recent perspective.^[^ ^22^ ^]^ Here, we aim to provide further insights into predicting actual ethanol performance when ethanol oxidation has occurred. For example, by utilizing the ethanol oxidation ratio of acetate to acetaldehyde observed in the control experiment (Figure 4b), which is ≈1.8 for K_2_CO_3_, we can roughly estimate the actual ethanol and acetate performance based on the measured acetaldehyde content at 500 mA cm^−2^ in Figure 1c. Under K_2_CO_3_ conditions, this approach suggests an increase in ethanol current density from 109 to 123 mA cm^−2^ and a decrease in acetate current density from 60 to 50 mA cm^−2^. It is important to note the uncertainty of this approach due to operational differences across various systems. Therefore, we encourage researchers to determine the corresponding values for their specific cases.

Overall, from an activity perspective, anodic oxidation impacts performance evaluation by causing an overestimation of the FE for acetate and acetaldehyde, while underestimating the FE for ethanol and propanol. It also reduces the cell voltage, leading to an overestimation of EE. Since anodic oxidation of liquid products is generally undesirable in COE, K_2_CO_3_ is a promising electrolyte for future applications for alcohol‐based production due to its greater resistance to alcohol oxidation and lower FE of H_2_ at large current densities.

### Electrolyte Effect on COE Durability

**Figure 5:**
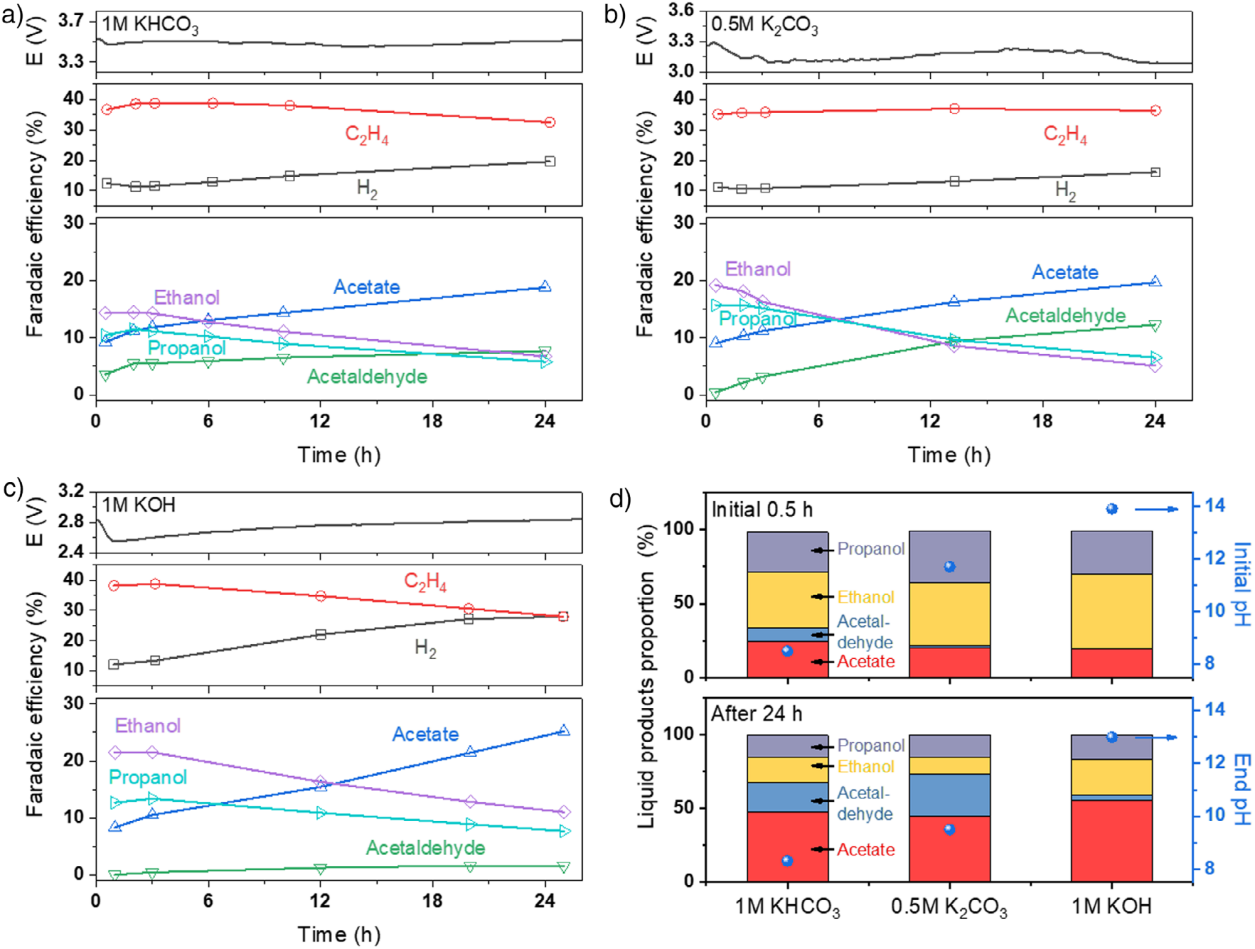
Changes of cell voltage, faradaic efficiency of different products (including hydrogen, ethylene, acetaldehyde, acetate, ethanol and propanol) in a) 1 m of KHCO_3_, b) 0.5 m of K_2_CO_3_ and c) 1 m of KOH at 300 mA cm^−2^ during 24 h COE; d) Comparison of liquid products proportion in different electrolytes and electrolyte pH at initial 0.5 and 24 h during COE period.

To investigate the influence of electrolytes on durability, 24 h COE experiments were conducted in different electrolytes with a 50 mL reservoir at 300 mA cm^−2^. A smaller reservoir was chosen over a larger one to amplify the effects of anodic oxidation within the electrolyte, including the impact of liquid product accumulation and the resulting pH changes. As shown in Figure 5a–c, the FE for C_2_H_4_ generally decreased over time, accompanied by an increase in the FE for H_2_ across all three electrolytes. Among them, 0.5 m K_2_CO_3_ exhibited the smallest decrease in FE_C2H4_ and the smallest increase in FE_H2_. As anticipated, liquid product distributions in all electrolytes changed significantly over time. Over the course of the electrolysis experiment, the production of ethanol and propanol gradually shifted toward acetate and acetaldehyde formation. The rates of acetate and acetaldehyde formation varied depending on the electrolyte, reflecting the oxidation kinetics of ethanol and propanol under different pH and with the liquid product accumulation.

Figure 5d provides further insights into the proportions of liquid products in different electrolytes and their corresponding pH values at 0.5 and 24 h. Initially, alcohol products (ethanol and propanol) dominated across all electrolytes, with slightly higher proportions in KOH. After 24 h, acetate became the major product (>50%) in all cases. Notably, KOH maintained a relatively higher ethanol proportion of 24%, while K_2_CO_3_ had the most acetaldehyde formation of any electrolyte, achieving a higher proportion of 28%. These results align with the oxidation trends of liquid products in different pH conditions. It is worth noting that the pH change of the anolyte is primarily driven by ethanol oxidation.^[^ ^27^ ^]^ The extent of pH change during electrolysis varies across different electrolytes due to their unique characteristics. For KOH, the pH decreased slightly from 13.9 to 13, remaining strongly alkaline, and thus it maintains a relatively low conversion of liquid products, consistent with control experiment observations. For K_2_CO_3_, the pH transitioned from weakly alkaline to more neutral (11.7–9.5), favoring acetaldehyde formation and its stabilization under these conditions.

These durability measurements highlight anodic oxidation drives dynamic changes in anolyte composition, disrupting pH and ionic balance, which in turn affects reaction kinetics and mass transfer. Significant variations in anolyte pH, even at the anode, can increase the risk of degradation for both anode and cathode catalysts. To mitigate anodic oxidation, it is essential to either develop efficient in‐line separation methods to extract liquid products from the anolyte or prevent liquid product crossover. Until now, the separation of different liquid products has remained costly and time‐consuming.^[^ ^31^ ^]^ For instance, separating acetaldehyde, ethanol, propanol, and acetate from a 0.5 m K_2_CO_3_ solution requires multiple steps. First, acetaldehyde, ethanol, and propanol can be separated via low‐temperature or fractional distillation. By carefully controlling the temperature, they can be isolated based on their boiling points. The concentrations of these compounds significantly affect the feasibility and efficiency of the distillation process. Following this, an acidification step is needed to isolate acetate and convert it into acetic acid. Since acetic acid has low solubility in certain organic solvents, such as ethyl acetate, it can be extracted using liquid–liquid extraction. From the perspective of preventing crossover, one promising direction is the elevated‐temperature COE, as higher temperatures can promote the evaporation of liquid products at the cathode,^[^ ^32^ ^]^ thereby preventing crossover. Additionally, advancements in membrane technology will be instrumental. To minimize liquid product crossover, membranes should possess low water content and small hydrophilic channel sizes to reduce free volume for unwanted diffusion, while still maintaining high hydroxide conductivity.^[^ ^15^, ^33^ ^]^ Moreover, the membranes should also be able to withstand elevated temperatures to allow for liquid products to come off as a vapor.^[^ ^34^ ^]^

Furthermore, acetaldehyde production from ethanol oxidation presents a novel research avenue. By optimizing reaction conditions (e.g., electrolyte), selective electrochemical oxidation of ethanol to acetaldehyde could provide a sustainable alternative to traditional oxidative dehydrogenation processes that require significant heating.^[^ ^35^ ^]^

## Conclusion

In this study, we investigated the influence of electrolytes on the selectivity of liquid products in MEA‐based COE using three electrolytes: KHCO_3_, K_2_CO_3_, and KOH. Our findings show that the electrolyte not only influences the cathodic CO reaction toward liquid products but also affects the reaction kinetics of the anodic oxidation of these products. Among the tested electrolytes, K_2_CO_3_ demonstrated the best potential for achieving high selectivity for high‐value products such as ethanol and propanol, due to its ability to maintain a moderate local alkaline environment and to resist anodic oxidation. Control experiments revealed that ethanol oxidation is much more severe compared to propanol oxidation in MEA‐based CO electrolysis. The highest reaction rate was observed in KHCO_3_, which is closely linked to its pH, resulting in significant formation of acetate and acetaldehyde. Durability measurements further underscored the critical importance of minimizing anodic oxidation to optimize MEA‐based COE for ethanol and propanol production. Potentially, the electrochemical oxidation of ethanol in neutral electrolytes could offer an alternative approach to acetaldehyde production. Our findings provide valuable insights into the performance trends of COE across different electrolytes with varying pH, highlighting the intricate relationship between reaction conditions and product selectivity.

## Conflict of Interests

The authors declare no conflict of interest.

## Supporting information



Supporting Information

## Data Availability

The data that support the findings of this study are available from the corresponding author upon reasonable request.
