# Supplementary material for: Electrolyte Effects in Membrane‐Electrode‐Assembly CO Electrolysis
Source: Angew Chem Int Ed Engl. 2025 Mar 27;64(22):e202501505. doi: 10.1002/anie.202501505 (PMC12105689; doi:10.1002/anie.202501505)
Supplement: Supplementary file 1 — Supporting Information [file ANIE-64-e202501505-s001.pdf]

Electrolyte Effects in Membrane-Electrode-Assembly CO Electrolysis

Qiucheng Xu[a,b], Bjørt Óladóttir Joensen[a], Nishithan C. Kani[a], Andrea Sartori[c], Terry Wilson[d], John R. Varcoe[d], Luca Riillo[e], Anna Ramunni[e], Jakub Drnec[c], Ib Chorkendorff[a], Brian Seger[a, *]

[a]*Surface Physics and Catalysis (SurfCat) Section, Department of Physics, Technical University of Denmark, 2800 Kgs. Lyngby, Denmark*

[b]*Laboratory of Inorganic Synthesis and Catalysis, Institute of Chemical Sciences and Engineering, Ecole Polytechnique Fédérale de Lausanne (EPFL), Lausanne CH-1015, Switzerland*

[c]*Experimental Division, European Synchrotron Radiation Facility, Grenoble, France*

[d]*Department of Chemistry, The University of Surrey, Guildford GU2 7XH, United Kingdom*

[e]*Industrie De Nora S.p.A., Via Leonardo Bistolfi 35, 20134 Milan, Italy*

**Corresponding author: Brian Seger, Email: brse@fysik.dtu.dk*

Part I: Experimental Procedures

1.1 Electrolyte and electrode preparation

The solution of KOH (1 M) or $KHCO_3$ (1 M) or K_2CO_3 (0.5 M) were prepared as anolyte. The chemicals including $KHCO_3$ ((99.7%, Sigma Aldrich), K_2CO_3 (≥99.95% trace metals basis, Sigma Aldrich), KOH (≥99.95% trace metals basis, Sigma Aldrich) were purchased and used without any further purification. Millipore-quality water (18.2 MΩ at 25°C) was used in all experiments. The cathode was prepared by spraying oxide-derived copper (OD-Cu) with loading of 1 mg cm^{-2} on the carbon-based gas diffusion layer (De Nora). A nickel foam (NF, SynLectro™) or a commercial IrO_2-based GDE (purchased from HPNow) was used as an anode for alkaline electrolyte and neutral electrolyte, respectively. A radiation-grafted anion-exchange membrane (AEM) containing N-methyl-piperidinium headgroups (called RG-MPIP, **Figure S10**) with thickness of 55 ± 5 μm and ion-exchange capacity (IEC) of 2.1 mmol g^{-1} was provided by Prof. John R. Varcoe's group.

1.2 Electrochemical cell assembly

A customized electrolyzer and the detailed MEA configuration as shown in **Figure S1** was used for the CO electrolysis. A RG-MPIP AEM was assembled between the cathode and anode with active electrode area of 1 cm^2. For each experiment, a fresh cathode, anode, and AEM was used. In a typical experiment, a dry CO (5N) was fed to the cathode GDE at a constant flow rate of 20 sccm by means of Vöegtlin red-y smart series mass flow controller. At the anode, different anolytes were circulated using a digital diaphragm pump (KNF NF 1.5TTDCB-4) which was set to 100% of its maximum current range (flowrate of *ca.* 50 ml/min). The outlet cathode gas stream was passed through a gas wash (20 ml pure water) to extract the liquid products and then went to gas chromatography (GC). The flow rate of the cathodic outlet streams of the electrolyzer was measured by the internal standard method (a constant flow rate of the inert N_2 gas sent into GC) or the volumetric flow meter (GFM Pro, Thermo Scientific). The flow diagram of the electrolysis setup is exhibited in **Figure S4**. At the end of each experiment, liquid samples were collected both at the anode and cathode. For the anolyte, we used a fixed anolyte volume of 200 mL and recycling it, except as otherwise stated.

To ensure reliability in our activity measurements (Figure 1), we used a relatively large

anolyte volume of 200 mL and completed the measurements within one and a half hours. Under these conditions, the electrolyte pH remained stable, with changes of no more than 0.1 units after electrolysis, suggesting that anodic oxidation remained relatively constant. To investigate the influence of electrolytes on durability (Figure 5), a smaller 50 mL reservoir was chosen over a larger one to amplify the effects of anodic oxidation within the electrolyte, including the impact of liquid product accumulation and the resulting pH changes.

1.3 Electrochemical measurements

All MEA tests were performed in the two-electrode system by using a potentiostat (Biologic VSP-89). All measured cell voltages were reported without any *iR* correction. Faradaic efficiency (FE) was calculated by equation as $FE_i = (n \cdot F \cdot C_i \cdot V)/I_{total}$, where n is the number of electrons transferred, F is Faraday's constant, C_i is the molar concentration of species i, V is the total volumetric flow rate, and I_{total} is the applied current at chronopotentiometry (CP) mode or the measured total current at potentiostatic mode. In principle, energy efficiency (EE) should be calculated using the FE at both the anode and cathode to determine the theoretical equilibrium potential. However, to highlight differences between electrolytes, the EE calculation in **Figure S9** was applied by using the water-splitting potential (1.23 V) as the equilibrium potential.

The polarization curves were measured by using a three-electrode setup, where NF (0.5 cm^2) was used as cathode, Ti mesh (4 cm^2) was used as anode and Ag/AgCl (1 M KCl) was as reference electrode. Overpotentials was corrected by the compensated resistance (R_u) that measured by performing an impedance at high frequency.

1.4 Gas and liquid products measurements

All gas products were analyzed using the GC (PerkinElmer Clarus 580 or Agilent 6890A) equipped with a thermal conductivity detector and a flame ionization detector. The liquid products were measured using an Agilent liquid chromatograph (1260 Infinity II) with 0.05 M H_2SO_4 as an internal eluent. For the 2-propanol experiment, liquid products were analyzed via 1H NMR, with 20 mM DMSO serving as the internal standard.

1.5 Synchrotron measurements

A customized synchrotron cell was used for the *operando* X-ray studies. A full description of synchrotron cell is provided in our previous work.[1] The wide-angle X-ray scattering (WAXS)

measurements were conducted at the ID31 beamline of the European Synchrotron Radiation Facility (ESRF) in Grenoble, France. The system was exposed to 68 keV X-rays with a beam size of 5 µm vertically and 20 µm horizontally parallel to the MEA in grazing incidence mode. The WAXS detector (Dectris Pilatus CdTe 2M) is positioned in the beam direction, 81 cm from the sample, to measure the scattered signal from the cell. The sample-detector distance and photon beam energy and detector tilt were calibrated using a Cerium dioxide (NIST) standard powder. The integration of the WAXS data is performed using pyFAI software package, by azimuthally integrating the ring intensity on the 2D detector to create the 1D WAXS pattern.

Part II: Supporting Figures

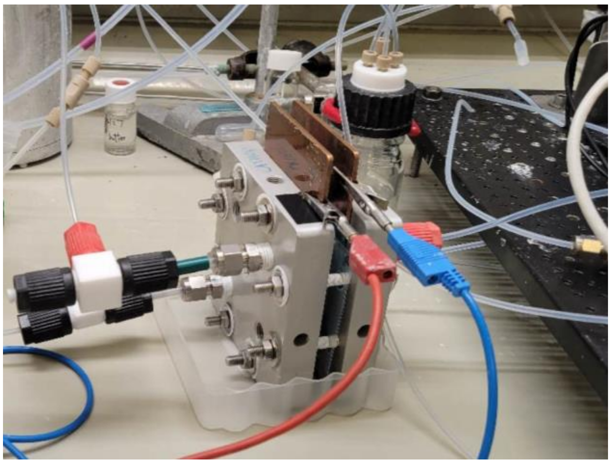

Figure S1. The digital image of customized electrolyzer.

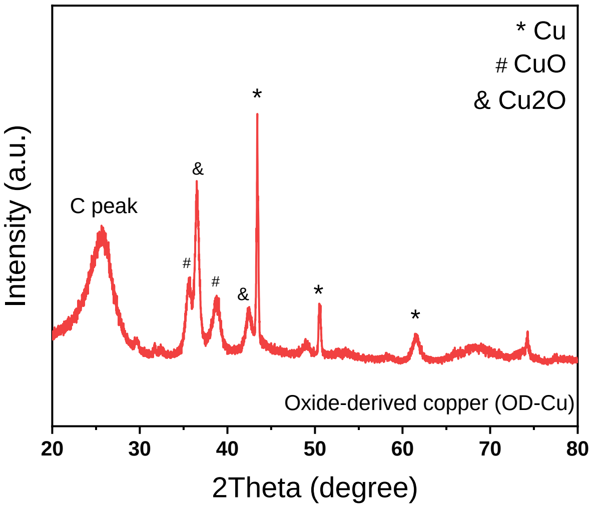

Figure S2. XRD Pattern of oxide-derived copper (OD-Cu) on the carbon gas diffusion electrode.

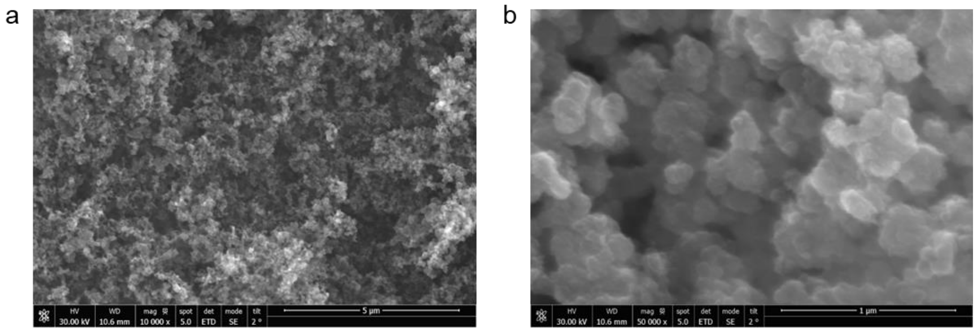

Figure S3. SEM images of OD-Cu on the carbon gas diffusion electrode.

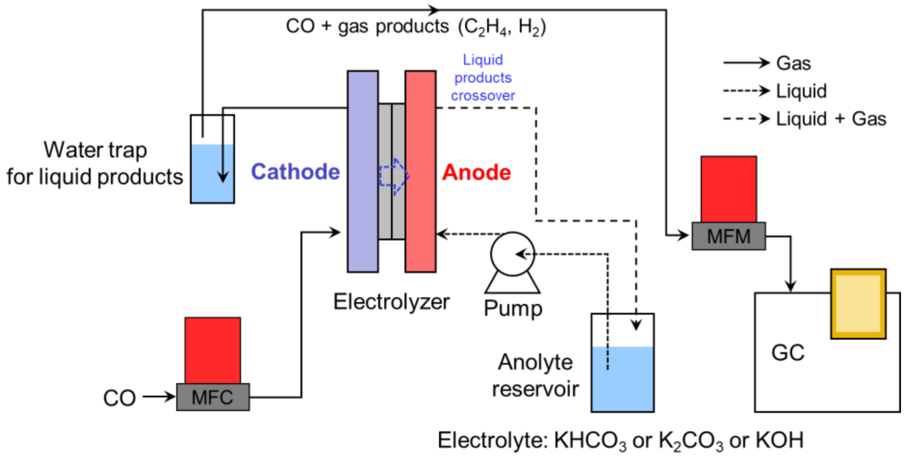

Figure S4. The flow diagram of the CO electrolysis setup. Blue arrow indicates the liquid products crossover. MFC: mass flow controller; MFM: mass flow meter; GC: gas chromatography

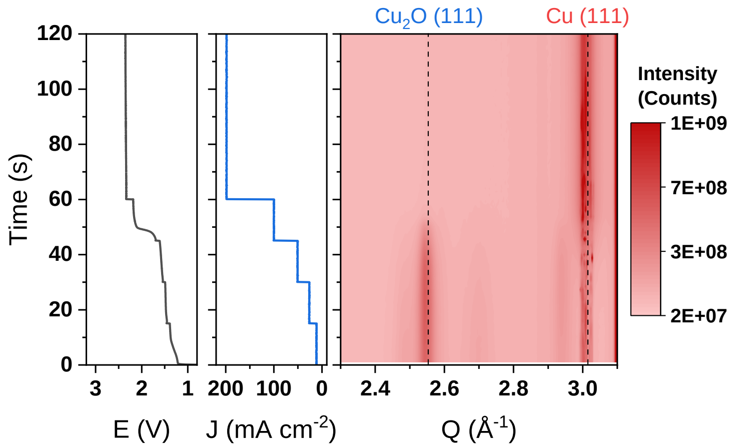

Figure S5. *Operando* WAXS at the region of catalyst layer inside the MEA during the CO electrolysis in 1M KOH. The Cu (111) peak's intensity increases with the decrease of Cu_2O (111) peak, suggesting the conversion from OD-Cu to metallic Cu.

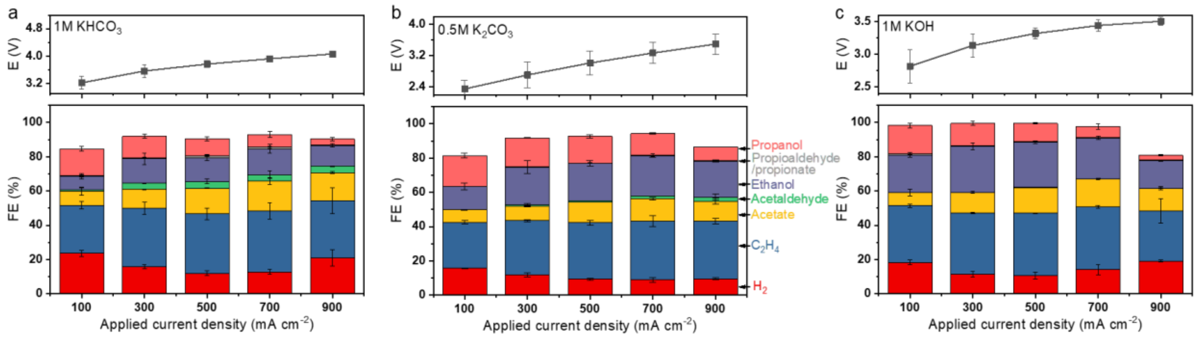

Figure S6. Cell voltage and FE of different products (including hydrogen, ethylene, acetaldehyde, acetate, ethanol and propanol) in (a) 1 M of $KCHO_3$, (b) 0.5 M of K_2CO_3 and (c) 1 M of KOH at variable applied current densities for CO electrolysis.

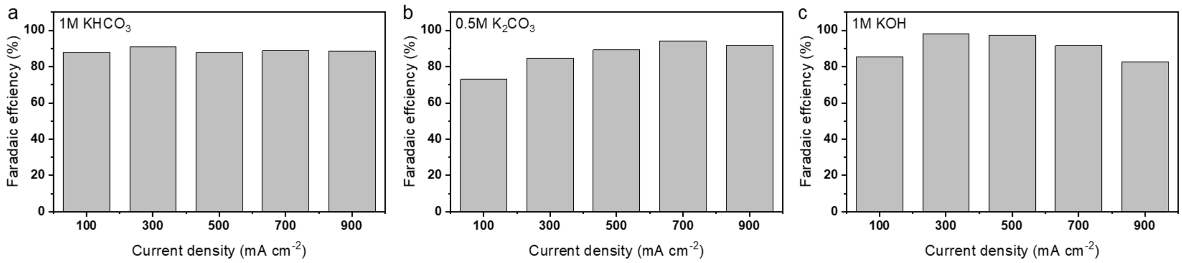

Figure S7. The FE of oxygen at the anode in (a) 1 M of $KCHO_3$, (b) 0.5 M of K_2CO_3 and (c) 1 M of KOH at variable applied current densities for CO electrolysis.

Notes: The gas products at the anode side were measured for one set of previous experiments. For K_2CO_3 and KOH, only O_2 was detected, with no measurable CO_2 signals. However, for $KHCO_3$, some CO_2 was detected. The absence of CO_2 in the KOH and K_2CO_3 cases suggests that CO_2 generated from the oxidation of liquid products is readily converted to HCO_3^- in the alkaline environment, preventing its detectable release as a gas. Additionally, CO_2 release in $KHCO_3$ electrolyte may originate not only from anodic oxidation but also from CO_2 neutralization, due to HCO_3^-/CO_3^{2-} crossover from the cathode. As a result, quantifying the exact amount of liquid products oxidized to CO_2 remains challenging. Moreover, the FE loss of O_2 follows a similar trend to the total FE loss at the cathode, indicating that the loss is primarily due to anodic oxidation of liquid products. However, we cannot determine whether this FE loss results from complete oxidation to CO_2 or partial oxidation.

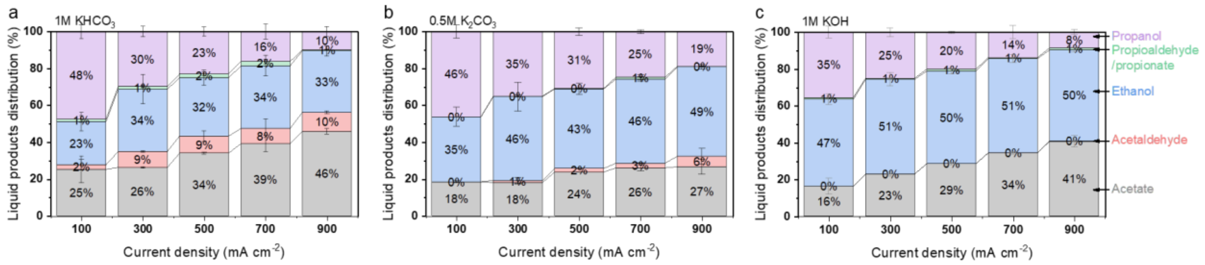

Figure S8. Liquid products distribution of different liquid products when operating COE in (a) 1 M of KCHO$_3$, (b) 0.5 M of K_2CO_3 and (c) 1 M of KOH at different current densities.

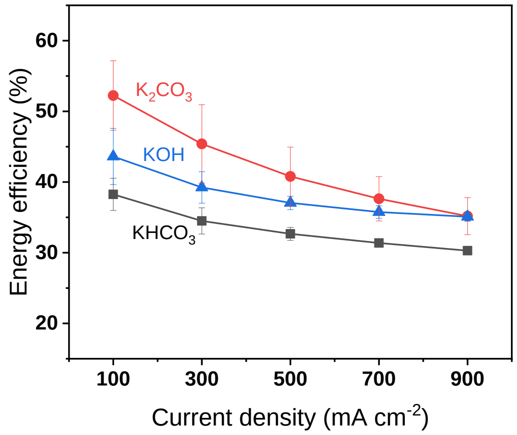

Figure S9. Energy efficiency at different electrolytes in terms of varying current densities.

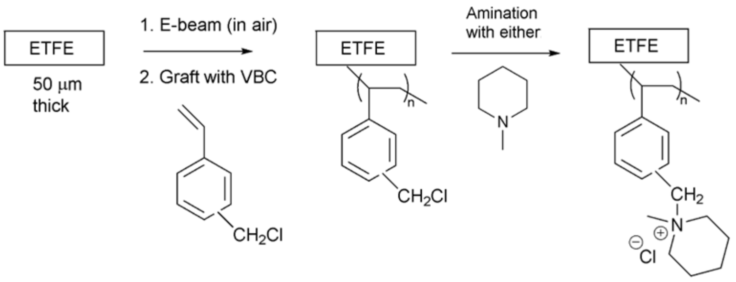

Figure S10. A summary of the synthesis process used to fabricate the MPIP-based RG-AEMs.

Notes: The membrane's backbone is composed of an ethylene-tetrafluoroethylene (ETFE) film, which has been radiation-grafted with vinylbenzyl chloride (VBC). Quaternary ammonium (QA) functional groups are introduced by reacting the grafted benzyl chloride ($-CH_2Cl$) groups with N-methyl-piperidine, forming N-benzyl-N-methyl-piperidinium cations. The membrane is initially synthesized in the chloride (Cl^-) form but can be converted to other anion forms, such as HCO_3^- or CO_3^{2-}, through ion exchange.

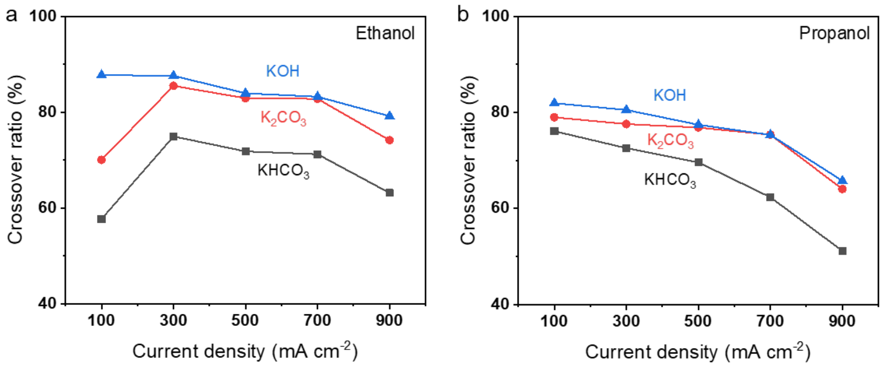

Figure S11. Crossover rate of (a) ethanol and (b) propanol when operating COE in (a) 1 M of $KHCO_3$, (b) 0.5 M of K_2CO_3 and (c) 1 M of KOH at variable applied current densities.

Notes: Both ethanol and propanol generally exhibit a similar trend, where the crossover ratio decreases with increasing current densities, except for ethanol at 100 mA cm^{-2} in K_2CO_3 and $KHCO_3$. These two outliers may be attributed to an unexpected high anodic oxidation, significantly affecting their amounts at the anode. This observation is consistent with their relatively low total faradaic efficiency (<90%) at 100 mA cm^{-2} (Figure S6). Furthermore, the crossover rate should be primarily governed by the concentration gradient between the anode and cathode and should theoretically increase with current density. However, the transport pathways within the membrane are limited in the actual reaction conditions, leading to a crossover saturation effect at higher current densities. Additionally, ethanol exhibits a slightly higher crossover ratio than propanol due to its smaller molecular size. The crossover ratios for different electrolytes, across all corresponding current densities, generally follow the trend: KOH > K_2CO_3 > $KHCO_3$.We hypothesize that this behavior is influenced by membrane swelling in different electrolytes with varying pH. Changes in electrolyte pH can induce membrane swelling, altering its nano/micro-pore structure and affecting the crossover of different molecules. For the MPIP membrane used in our study, swelling follows the trend OH^- > CO_3^{2-} > HCO_3^-,[2] agreeing with the observed trend of ethanol and propanol crossover.

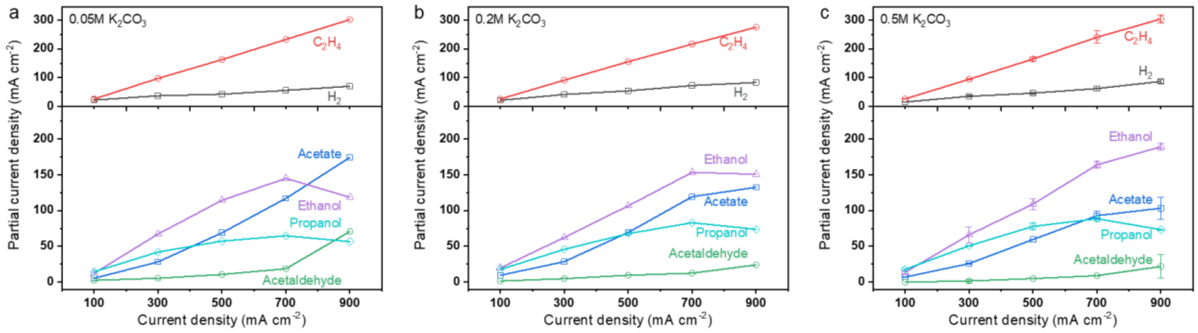

Figure S12. Partial current densities of different products (including hydrogen, ethylene, acetaldehyde, acetate, ethanol and propanol) when operating COE in (a) 0.05 M, (b) 0.2 M and (c) 0.5 M of K_2CO_3.

Notes: Minimal differences were observed as a function of electrolyte concentration, though a slight shift towards more oxidized products (acetate and acetaldehyde) was noted at lower concentrations and higher current densities (>700 mA cm^{-2}), suggesting that anodic oxidation becomes more pronounced under these conditions. This effect may be attributed to the steric hindrance of anions in the electrolyte, where the accumulation of anions near the anode surface reduces the diffusion of neutral molecules like ethanol and propanol, thereby mitigating the oxidation of liquid products.

Reference

1. Moss, A.B., Hätinen, J., Kúš, P., Garg, S., Mirolo, M., Chorkendorff, I., Seger, B., and Drnec, J. (2023). Versatile high energy X-ray transparent electrolysis cell for operando measurements. J. Power Sources 562, 232754.
2. Willson, T.R., Rodriguez, C.A.G., Xu, Q., Frow, J., Foglia, F., Smith, K., Ravikumar, R., Vinothkannan, M., Mahmoudi, N., Salam, I. Periasamy, A.P., Whelligan, D. K., Mamlouk, M., Lin, H., Seger B., and Varcoe, J. R. (2023). Radiation-grafted anion-exchange membranes for CO_2 electroreduction cells: an unexpected effect of using a lower excess of N-methylpiperidine in their fabrication. Journal of Materials Chemistry A, 11(38), 20724-20740.
